# Malignant hypertension secondary to iliac artery stenosis after kidney transplantation: a case report

**DOI:** 10.11604/pamj.2021.40.132.30927

**Published:** 2021-11-03

**Authors:** Imtinen Ben Mrad, Lilia Ben Fatma, Ben Mrad Melek, Ikram Mami, Boutheina Ben Abdellatif, Rim Miri, Sobhy MleyhiKarim Zouaghi, Raouf Denguir

**Affiliations:** 1 Cardiology Department, Hbib Thameur Hospital, Tunis, Tunisia,; 2 Nephrology Department, Rabta Hospital, Tunis, Tunisia,; 3 Cardiovascular Surgery Department, Rabta Hospital, Tunis, Tunisia

**Keywords:** Hypertension, renal impairment, kidney transplantation, endovascular, case report

## Abstract

Iliac artery stenosis is a rare complication after renal transplantation. This complication affects elderly patients and related to atheromatous disease. It mimics the same clinical presentation as a transplant renal artery stenosis or renal artery stenosis. This entity is can be responsible for serious complications such as renal dysfunction, malignant hypertension and acute pulmonary oedema. We present in this paper the case of a 51-year-old patient, who benefited 7 years early of renal transplantation, with a good initial result, and who was admitted actually for malignant hypertension and renal function impairment due to an iliac artery stenosis proximal to the renal transplant and who was treated with a stenting angioplasty of the external iliac artery with a mixed outcome. Our case highlights the importance of the early diagnosis and treatment of such complications to avoid definitive renal failure and permanent hypertension.

## Introduction

End-stage stage disease is a public health problem in Tunisia [1]. The number of kidney transplantation performed in our country is growing exponentially with expanding indications, even in elderly patients [1]. As a result, vascular complications after kidney transplantation are becoming more common. These complications can affect the renal graft artery or the donor iliac artery. These types of complications need to be diagnosed and treated early as they can jeopardize the viability of the graft and can be responsible for serious complications such as renal dysfunction, malignant hypertension and acute pulmonary oedema [2]. We present in this paper the case of a 51-year-old patient, who benefited 7 years early of renal transplantation, with a good initial result, and who was admitted actually for malignant hypertension due to an iliac artery stenosis proximal to the renal transplant and who was treated with a stenting angioplasty of the external iliac artery with a mixed outcome.

## Patient and observation

**Patient information:** a 51-year-old man who had been diagnosed with end-stage renal disease in 2012 requiring hemodialysis and who had a living donor kidney transplantation in 2013 was admitted to the nephrology department for an acute renal failure and refractory hypertension. His anamnesis was also remarkable for a history of heavy smoking well before and after kidney transplantation. He had an angioplasty of the left superficial femoral artery three years earlier for critical limb ischemia. The patient complains also for a mild claudication of the right limb with a walking perimeter equal to 600 meters.

**Clinical findings:** the physical examination showed diminished femoral and distal pulses in the right side. The ankle brachial index was 0.7. The patient´s blood pressure was 190/110 mmHg despite taking four antihypertensive drugs.

**Diagnostic assessment:** the biological examinations showed a high level of creatinine at 370 mg/dL with a calculated clearance 25 ml/min, a potassium level at 5.5 mmol. A color Doppler ultrasound showed a significant stenosis in the right external iliac artery, just proximal to the anastomosis of the renal graft.

**Figure 1:**
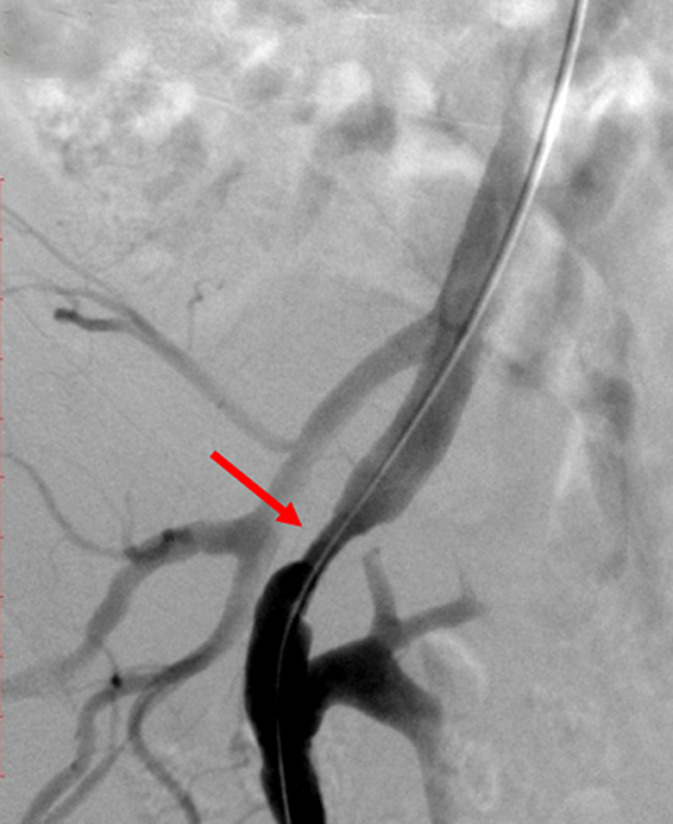
angiogram showing a tight stenosis of the external iliac artery 1 cm just proximal to the anastomosis of the renal transplant (red arrow)

**Figure 2:**
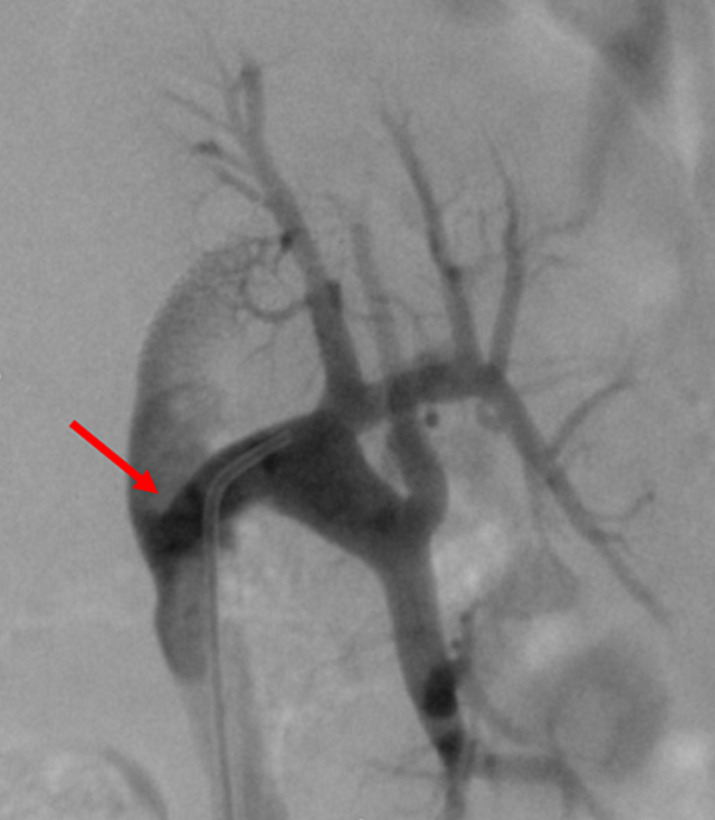
angiogram showing a patent renal transplant anastomosis without stenosis (red arrow)

**Figure 3:**
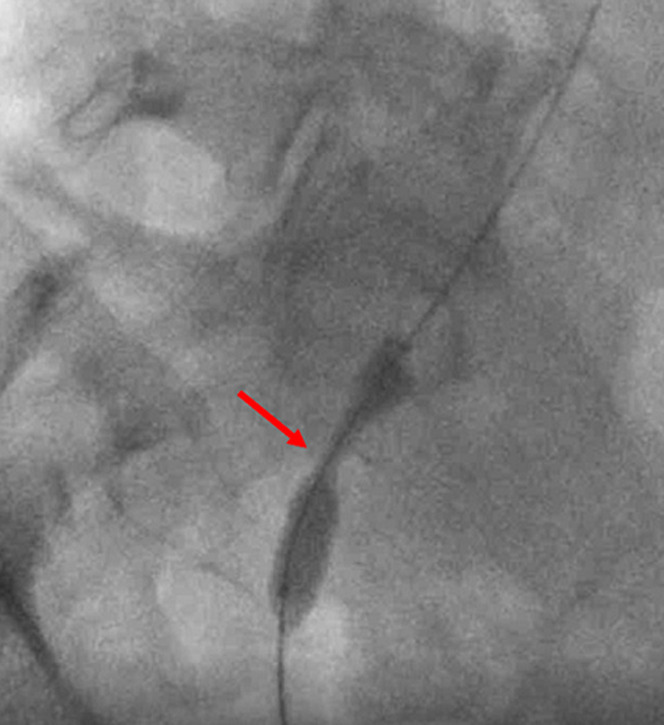
angiogram showing stent deployment in the external iliac artery (red arrow)

**Figure 4:**
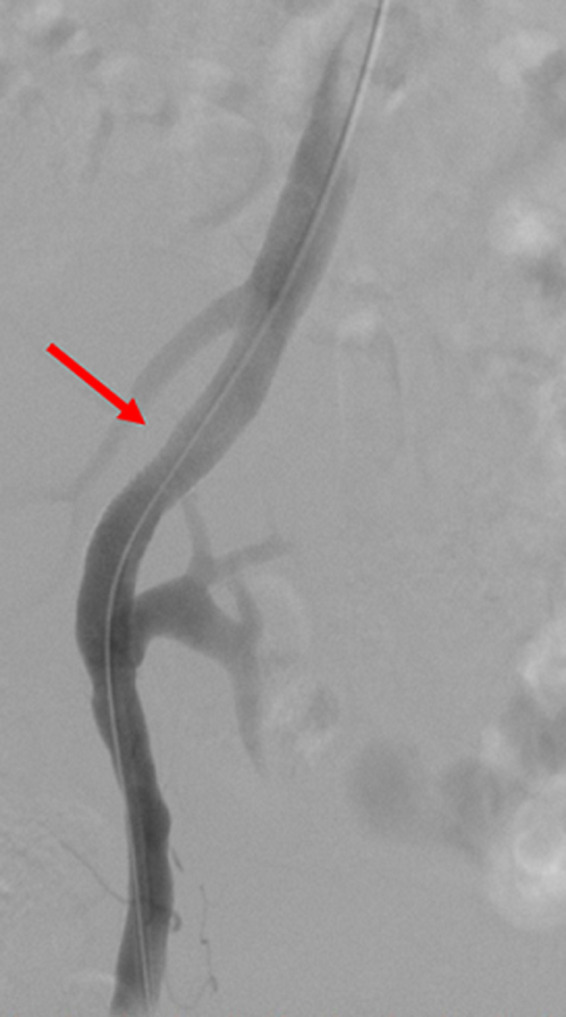
final angiogram showing a patent iliac artery after stenting without residual stenosis (red arrow)

**Therapeutic intervention:** in order to improve kidney function and high blood pressure we decided to treat this iliac artery stenosis. After a retrograde right femoral artery puncture, an 11 cm 6F vascular Sheath was placed. First angiogram showed a tight stenosis of the external iliac artery 1 cm just proximal to the anastomosis of the renal transplant (Figure 1). This latter was patent, without any stenosis (Figure 2). The lesion was crossed easily with a 0.035 hydrophilic guidewire. Percutaneous transluminal angioplasty (PTA) was successfully performed with stenting of the lesion by a 7mm diameter 37 mm length Express balloon expandable Stent (Boston Scientific) (Figure 3). Final angiography showed a good result (Figure 4).

**Follow-up and outcomes:** three days after this procedure, the patient presented left chest pain with troponin elevations and shifting in pre-ECG territory. Emergency coronary artery was objectified of bi-trunk lesions on the IVA and right coronary artery. The patient has a coronary angioplasty with a good result. After a 6-month follow-up period, despite the success of angioplasty and the disappearance of claudication symptomatology, the patient kept a mild hypertension with blood pressure at 145/80 mmHg under only calcic inhibitor, with a partial amelioration of the kidney function, biological control showed a creatinine level at 160 mg/dL. A US duplex showed a patent iliac stent.

**Informed consent:** informed consent was obtained from the patient for this publication.

## Discussion

Iliac artery stenosis (IAS) is a rare complication after renal transplantation [3]. The prevalence of IAS during fellow-up after a renal transplantation varies from 0.37% to 1.5% [3,4]. This complication is frequent in elderly patients related to atheromatous disease. We thought that arterial clamping at the time of the kidney transplantation might be an associated factor with the development of these lesions. In our case, it was a diffuse atheromatous disease, as evidenced by the coronary artery disease. It mimics the same clinical presentation as a transplant renal artery stenosis or renal artery stenosis [5]. This entity is a known cause of renal graft dysfunction and/or increased hypertension [6]. Sometimes it´s responsible like in our case of vascular claudication [7], and in extreme cases of a flare-up of acute pulmonary oedema and diuretic congestive heart failure [8]. It´s is associated with poorer graft survival [9].

The duplex US is the best exam to make the diagnosis in such cases, CT scan must be avoided to prevent impairment of kidney function secondary to the use of the contrast product. Endovascular management of these IAS must be the first line treatment strategy, it is a simple procedure but we must be careful not to cover the origin of the renal graft artery. However, the diagnosis of these lesions must be early to avoid impairment kidney function and malignant hypertension, which can be irreversible if treated late. In our case, despite the angiographic success, and the amelioration of the hypertension, we failed to preserve total renal function because the diagnosis was made late. Monitoring during follow-up of a patient with a kidney transplant should include palpation of the peripheral pulse to detect this type of arterial lesion, and if there is any doubt, a Duplex US can do the diagnosis.

## Conclusion

Renal transplantation is considered as an optimal treatment for end-stage renal disease. However, complications occur in both the immediate postoperative period and later. Iliac artery stenosis after transplantation certainly is an uncommon complication but it represents a cause of renal graft dysfunction and uncontrolled hypertension. Endovascular treatment is a noninvasive option, which can help obtain renal graft salvage, and hypertension stabilization provided it be done early.
